# Supplementary material for: Spatial soft sweeps: Patterns of adaptation in populations with long-range dispersal
Source: PLoS Genet. 2019 Feb 11;15(2):e1007936. doi: 10.1371/journal.pgen.1007936 (PMC6386408; doi:10.1371/journal.pgen.1007936)
Supplement: S1 Appendix — Detailed descriptions of analytical results and approximations used in the text, verified by additional simulation results. Includes analysis of preliminary simulation results for planar (2D) ranges. (PDF) [file pgen.1007936.s001.pdf]

S1 Appendix to *Spatial soft sweeps: patterns of adaptation in populations with long-range dispersal*

Jayson Paulose,[1,2] Joachim Hermisson,[3] and Oskar Hallatschek[1]

[1] *Departments of Physics and Integrative Biology, University of California, Berkeley, CA, USA*

[2] *Department of Physics and Institute of Theoretical Science, University of Oregon, Eugene, OR, USA*

[3] *Department of Mathematics and Max F. Perutz Laboratories, University of Vienna, Austria*

A. FORMS FOR $\ell(t)$ AND χ

Here we describe the analytical forms for $\ell(t)$ used to compute the predictions for the characteristic length scale χ in main text Fig. 4. Ref. 1 derived asymptotic growth forms for the long-time limit of the domain core $\ell(t)$ (i.e. the region within which the occupancy of the range by an isolated domain is of order 1) for dispersal kernels with tails that fall off as $r^{-(\mu+d)}$:

$$\begin{aligned} &A\exp(Bt^{\eta}), && 0<\mu<d,\\ &A\exp\left[\frac{\log^2(Bt)}{4d\log 2}\right], && \mu=d,\\ &At^{1/(\mu-d)}, && d<\mu<d+1,\\ &At\log(Bt), && \mu=d+1,\\ &At, && \mu>d. \end{aligned} \tag{A1}$$

Here, $\eta=\log_2[2d/(d+\mu)]$, and A and B are magnitude scales for ℓ and t that depend on μ and on details of the dispersal kernel. (In the wavelike growth regime, $\mu>d$, A is the front velocity of the growing domain.) The logarithmic correction to linear growth for $\mu=d+1$ is a conjecture for $d=2$, which is supported by simulation data.

To extract A and B for the specific kernels used here, we performed separate simulations in which domains were grown from a single seed at the origin at $t=0$. The domains were grown up to final masses of order 10^8 for $\mu\le 1$ and 10^5 for $\mu>1$ in 1D, and of order 10^7 in 2D, with the background mutation rate turned off. For each value of μ, 20 independent simulations were performed and the mass evolution over time, averaged over the independent runs, was equated to $\omega_d\ell^d(t)$ following our definition of $\ell(t)$ in the main text. The $\ell(t)$ thus extracted was fit to the growth forms to obtain A and B. (Given that the growth of ℓ with t can be extremely fast for $\mu<d+1$, in practice we fit the functional dependence of $\log\ell(t)$ against $\log t$, with $\log A$ and $\log B$ as free parameters.) Using the total mass as a proxy for $\ell(t)$ leads to an overestimate of the true size of the core, because it also counts individuals in the inevitable "halo" that exists due to jumps from the core to regions outside it during the stochastic growth process. The halo contains a fraction of the individuals in the core, which falls as μ increases. This correction is expected to provide a multiplicative constant of order 1 to $\ell(t)$, which is inconsequential to the prediction of $X_{\rm ave}$ which itself equals χ only up to an overall constant for each μ.

The asymptotic forms only agree with the measured single-allele growth profiles when $\ell(t)$ has grown beyond a certain size. However, this threshold size becomes extremely large (i.e. order of the simulation range or larger) for values of μ close to d [1], making the asymptotic forms of limited utility to predict χ. Ref. 1 also derives an analytical scaling form for the behaviour of $\log_2\ell(t)$ over a much broader range of times for μ close to d, which reads

$$\log_2\ell(t)\approx\log A+\frac{2d}{\delta^2}\left[(Bt)^{\zeta}-\zeta\log(Bt)-1\right], \tag{A2}$$

where $\delta=\mu-d$ and

$$\zeta=-\frac{\delta}{2d\log 2},\qquad \delta>0,$$

$$\zeta=-\frac{\log(1+\delta/2d)}{\log 2},\qquad \delta<0.$$

As before, we used fits of $\log\ell(t)$ against $\log t$ to obtain the parameter values $\log A$ and $\log B$. From our fits to the single-allele growth simulations, we found that the scaling form is significantly more accurate than the asymptotic forms of Eq. A1 for $\mu\le 1.4$ in 1D, and $\mu\le 2.6$ in 2D (except fo the marginal value $\mu=d$ in each case). As a result, we use the scaling form for our predictions of χ for these values of μ. Table I summarizes the values of $\log A$ and $\log B$ extracted from fits to the theoretical forms in Eqs. A1 and A2 as appropriate.

In all cases, the forms for $\log\ell(t)$ with fitted values for A and B are accurate to within a few percent for $\ell(t)$ of order 20 and larger. The inaccuracy of $\ell(t)$ for smaller domains leads to discrepancies between the measured average clone size and the prediction based on χ^d for large μ and high rescaled mutation rates, which drive down the average clone extent into the regime of inaccurate $\ell(t)$.

Once A and B are determined from the fit either to Eq. A1 or Eq. A2, the relation defining the characteristic length, Eq. 1 (main text), is solved to obtain $t^*(u)$ and $\chi_\mu(u)=\ell_\mu(t^*)$. Table 1 in the main text reports the functional forms for χ derived upon assuming that $\ell(t)$ follows the asymptotic forms. When the more complex scaling form is used for $\ell(t)$, Eq. 1 in the main text can still be solved to obtain an analytical solution for $\chi(u)$ in terms of Lambert W-functions. For each dispersal kernel, the solution $\chi_\mu(u)$ is analytically determined taking only μ, and the values of A and B estimated from fits (as reported in Table I) as inputs.

The characteristic length scale χ quantifies the balance between domain growth and mutations that sets

1D simulations			2D simulations		
μ	$\log A$	$\log B$	μ	$\log A$	$\log B$
0.2*	0.122	0.270	0.5*	−0.333	0.403
0.4*	−0.146	0.509	1.5*	−0.788	1.31
0.6*	0.274	0.671	2.0	−1.26	2.22
0.8*	0.417	0.861	2.2*	−1.76	2.72
1.0	0.0246	1.23	2.4*	−2.17	3.32
1.2*	−0.242	1.40	2.5*	−3.17	4.23
1.4*	0.302	1.32	2.6*	−4.09	5.21
1.6	−0.841	na	2.8	−0.489	na
1.8	0.558	na	3.0	−1.10	0.142
2.0	0.0253	0.00	3.5	0.271	na
3.0	0.00	na	4.5	−0.002 96	na
4.0	−0.271	na	5.5	−0.105	na

TABLE I. **Values of parameters A and B from fits.** Estimates of $\log A$ and $\log B$ obtained by fitting the growth dynamics of single clones as described in the text. The asterisk denotes use of the scaling form (Eq. A2) over the asymptotic form (Eq. A1).

the average domain size via $X_{\text{ave}} \propto \chi^d$ up to a multiplicative constant of order 1; the precise relationship between χ^d and X_{ave} is determined by the distribution of domain sizes about the characteristic size, which is in turn established by the complete growth dynamics. We have an explicit form for the domain size distribution in the constant-velocity wavelike growth regime in 1D, $\mu > 2$ (Eqs. A5 and A6), which allows us to derive $X_{\text{ave}} = 2\sqrt{2/\pi}\chi \approx 1.6\chi$ in this regime. For the 1D results in Fig. 4, we find that multiplicative constants close to 1.6 also lead to agreement between $X_{\text{ave}}(u)$ and $\chi(u)$ for other values of μ, over many orders of magnitude of u. The agreement is weakest for high u which corresponds to small domains (average clone sizes of 100 or smaller); here the functional forms of $\ell(t)$ are least accurate and stochastic effects begin to dominate the deterministic growth implied by $\ell(t)$.

B. SIMULATION RESULTS IN 2D

Here, we describe preliminary results for average clone mass, clone extent, and frequency spectra as measured from 2D simulations. Simulating large ranges is a challenge in two dimensions: effectively simulating a system in which key jumps are of order l in length requires a range with over l^2 demes (in contrast to l demes in 1D). We have succeeded in simulating ranges of linear size $L = 4096$ (hence $4096^2 \approx 1.6\times10^7$ demes), and restricted ourselves to a range of mutation rates for which the total range mass is many times the average clone mass, so that we are in the regime of multiple-origin sweeps. However, we still expect finite-size effects to be significant for measures that depend on the spatial extent of the halo, which can stretch out to many times the mass-equivalent radius for small μ.

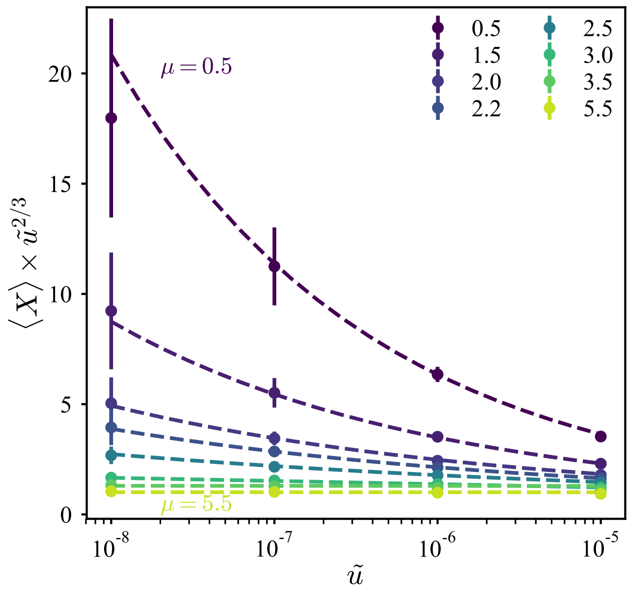

FIG. A1. **Average clone mass and mutation-expansion balance in 2D simulations.** The average clone mass measured from 2D simulations as a function of rescaled mutation rate, scaled by the expected dependence ($\propto \tilde{u}^{2/3}$) for wavelike growth. Each point represents an average over 48 independent simulations and error bars denote measured standard deviations across repetitions. Dashed lines show the theoretical prediction $\pi\chi^2$, using $\chi = \chi_\mu(\tilde{u})$ functions described in Appendix A. Each theory line is multiplied by a μ-dependent magnitude factor whose value is 0.8 for $\mu < 3$, 0.75 for $\mu = 3$, and 0.73 for $\mu > 3$.

Fig. A1 compares the average clone size to the theoretical expectation $\pi\chi^2$, where the functions $\chi_\mu(\tilde{u})$ are described in Appendix A. As with the 1D results, we find quantitative agreement with the theory lines upon using a single additional parameter — an overall magnitude scale which varies between 0.75 and 0.8.

Fig. A2 reports the spatial extent of the clones from the two largest mutation rates, for which finite size effects are smallest. In 2D, we define the extent in terms of the eighth central moment: $r_{\text{max}}^8 \equiv \sum_{i=1}^{X} |\mathbf{r}_i - \mathbf{r}_{\text{cm}}|^8/X$, where i indexes the demes belonging to that clone, $\mathbf{r}_i$ is the position vector of deme i (computed modulo $L/2$ for each component to account for periodic boundary conditions), and $\mathbf{r}_{\text{cm}} \equiv (\sum_{i=1}^{X} \mathbf{r}_i)/X$ is the clone center of mass. The use of a high moment in the definition of r_{max} ensures that the farthest demes from the centre of mass contribute strongly to r_{max} even if they are rare. The specific choice of the eighth moment balances the need to emphasize the farthest demes (which favours a high moment) with the necessity of preventing loss of floating-point precision in the computation (which requires that

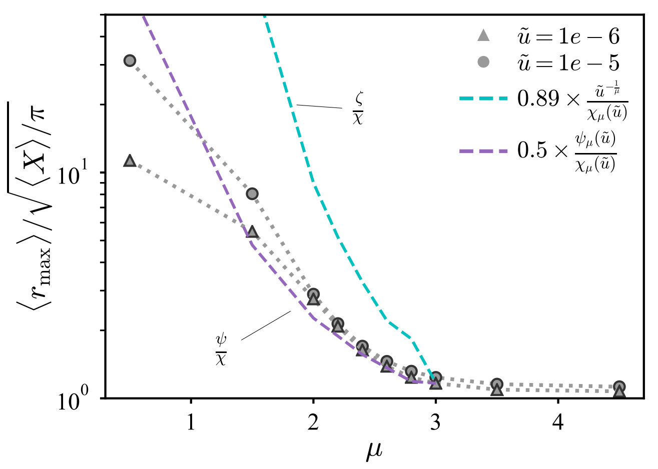

FIG. A2. **Spatial extent of clones in 2D simulations.** Ensemble-averaged spatial extent of clones in 2D, normalized by the ensemble-averaged mass-equivalent radius. See text for definition of $r_{\max}$ in 2D. Dashed lines show theoretical expectations ζ/χ and ψ/χ for $\tilde{u} = 1e-6$, computed as described in Appendix A. The prefactor was chosen so that the lines coincide with the simulation data point at $\mu = 3$. Finite size effects are more severe in 2D, and the measured values for $\mu < 2$ underestimate the true values that would be measured in an infinitely large range.

the moment not be too high). Using the sixth moment leads to similar results. By contrast, using too low a moment (such as the second moment, which provides the radius of gyration of the clone) gives values of $r_{\max}$ that are very close to r_{eq} since the core provides the major contribution.

We find that the dependence of the ensemble-averaged extent on the dispersal kernel is well captured by the length scale ψ in the regime of power-law growth in 2D, $2 < \mu < 3$, with a single additional parameter setting the overall magnitude scale. We note that the asymptotic ratio $\psi_{\text{as}}/\chi_{\text{as}}$, which was successful in reproducing $r_{\max}$ for the 1D data, does *not* agree with the 2D simulation data for the current parameter range. This is because the typical sizes of clones in the 2D simulations is too small for the asymptotic growth rule ($\ell(t) \sim t^{1/(\mu-d)}$) to be accurate. Instead, the scaling solution from Appendix A, which accurately captures the growth of single clones at the relevant size scales, must be used.

As was seen with the 1D data, the extent starts to depart from ψ as $\mu \to d$, consistent with an increased prominence of rare jumps out of the core region that land beyond well-established satellite clusters. However, the measured extent remains far below the theoretical bound ζ/χ, which grows extremely fast as μ falls below 2. We hypothesize that the ensemble averages are severely limited by finite-size effects; to attempt to match the theoretical expectation for $\mu = 1$, for instance, we would require range sizes over an order of magnitude larger in linear size, beyond our current capabilities for 2D simulations. Nevertheless, our limited simulations confirm that clones can attain a spatial extent many times larger than their mass-equivalent radius as the dispersal kernel is broadened.

To summarize, the results from preliminary 2D simulations show quantitative evidence for the relevance of the length scale χ, when combined with theoretical predictions for $\ell(t)$ from Ref. 1. The simulations also show that the halo can extend over much longer distances than expected for compact clone, with evidence for the relevance of the length scale ψ obtained from the core-halo picture in the power-law growth regime $d < \mu < d + 1$. However, more extensive simulations with much larger range sizes are needed to quantitatively test the relevance of the second scale ζ.

C. ALTERNATIVE DERIVATION OF SECONDARY LENGTH SCALE ψ

Here, we provide an alternative estimate for the length scale ψ that sets the extent of the halo of a "typical" clone, which agrees with the estimate $\psi = \ell(2t^*)$ proposed in the main text. The iterative scaling picture of Ref. 1 argues that, for growth in the marginal regime near $\mu = d$, key jumps that land at a distance $\ell(t)$ from the mutational origin typically occurred around time $t/2$ and spanned a distance of roughly $\ell(t)$ connecting source and target regions each of size $\sim \ell^d(t/2)$ (Fig. 2b). The core extent at a given time constrains the expected number of these key jumps that have contributed to the core boundary by that time: they can be neither too rare (in which case the core would not have reached the purported boundary) nor too common (which would imply that the region should have been filled much earlier). Since the number of key jumps is itself set by the extent of the core (the source for the jumps) together with the jump kernel, the above constraint equates to a self-consistency requirement on $\ell(t)$ [1]:

$$t\,\ell^{2d}(t/2)\,G[\ell(t)] \sim 1,$$

where $G(r) = J(r)r^{1-d}/\omega_d$ is the rate of jumps per unit area of source and target regions when both are separated by a distance r. In the soft-sweep model, key jumps compete with new mutations in the target region, which occur at a rate of order $\tilde{u}\ell^d(t/2)$. The growth of the halo is obstructed by new clones when the rate of mutations arising in the target region becomes comparable to the rate of key jumps into it from the expanding core. This requires

$$\tilde{u}\ell^d(t/2) \sim G[\ell(t)]\ell^{2d}(t/2) \sim 1/t \Rightarrow t\ell^d(t/2) \sim 1/\tilde{u}. \tag{A3}$$

Up to factors of order unity, the above scaling relation is satisfied by $t = 2t^*$, where t^* was the solution to Eq. 1. Therefore, we arrive at the same expression, $\psi \equiv \ell(2t^*)$, for the characteristic halo extent as we had derived in the main text from considerations of the jump-driven growth of *unobstructed* clones.

D. EXACT ALLELE FREQUENCY SPECTRA IN THE PANMICTIC AND 1D WAVELIKE SPREADING LIMITS

i. Panmictic limit

The panmictic limit in our lattice model would correspond to jumps being attempted from the source deme to a randomly chosen deme in the entire range. The allele frequency spectrum and related sampling probabilities can be computed exactly in this limit by mapping to an urn process. To see this, consider the evolution of allele frequencies in our lattice model when the fraction of wildtype sites is w and mutants occupy the lattice with individual fraction f_i for mutant i. At the next time step, the probability weight associated with picking a wildtype site to introduce a new mutation is $\tilde{u} \times Nw = \theta w$, where $\theta = \tilde{u}N$ is the initial mutation rate for the empty lattice. By contrast, the probability weight associated with picking a site of mutant type i for an attempted dispersal event is Nf_i, but only a fraction w of these attempted dispersal events is successful since the mutant only fixes in the target deme if it contains the wildtype. Therefore the probability weight of a successful reproduction of mutant i is Nwf_i. The final statistics of clone sizes is determined by the *relative* rate of mutation to reproduction at each time step [2] (unlike the times for the appearance of new clones which depends on the absolute rates), which is θ versus $n_i = Nf_i$ at all times since the wildtype fraction drops out.

The genealogy of new mutants in this model is identical to that of a stochastic process called Hoppe's urn [3], which begins with an urn containing a single black ball with an assigned probability weight θ. At any time step, a ball is picked from the urn with probability proportional to its weight. If the black ball is chosen, it is returned along with a ball with a new colour and probability weight 1 (a new mutant). If a coloured ball is chosen, it is returned along with one copy of itself. The relative rate of mutation to the duplication of a ball with colour i is θ versus n_i at each turn, thus establishing the equivalence to our lattice model. The distributions of mutant frequencies in this urn model are the same as those for the infinite allele model at equilibrium [4]. In particular, the allele frequency spectrum is

$$f_\infty(x) = \frac{\theta}{x}(1-x)^{\theta-1}. \tag{A4}$$

Fig. 6 shows that panmictic simulations reproduce the theoretical limit, which also persists for $\mu \approx 0.5$ in two dimensions.

The average clone size in the panmictic limit can be obtained from the allele frequency spectrum by computing the expected number of distinct clones n_c. The smallest possible clone frequency is $1/N$. Therefore, the expected number of distinct clones, n_c, is the sum of all allowed allele frequencies, i.e. $n_c = \int_{1/N}^{1} f(x)\,dx$, which can be evaluated exactly using $f(x)$ from Eq. A4. For large N, we have $n_c \approx \tilde{u}[-1+\theta+N\log N - N(\gamma+\psi_0(\theta)]$, where γ is the Euler-Mascheroni constant and ψ_0 is the digamma function. A further simplification, valid for $\theta \gg 1$, is $n_c \approx \theta \log(1/\tilde{u})$ [5]. Once n_c is computed, the average clone size is N/n_c.

Note that a mapping of the parallel adaptation model to an urn process was also identified in preprint [6].

ii. Wavelike spreading limit in 1D

For $\mu > d+1$, domains are predicted to grow in radially expanding waves, whose speed depends on the details of the dispersal kernel. The statistics of soft sweeps in this limit was previously explored by Ralph and Coop [7], who observed the equivalence of the process in the wavelike limit to Kolmogorov-Johnson-Mehl-Avrami (KJMA) models of grain growth. KJMA models track the evolution of isotropic domains which nucleate at random positions in space at a constant rate. Nucleated domains grow isotropically at a constant front velocity until they run into other domains, leaving a boundary separating domains that nucleated at different origins. The final pattern of domains matches the spatial pattern of clones in the mutation-expansion model, where individual domains correspond to distinct mutants.

In one dimension, the final grain size distribution for a KJMA process in which each nucleation gives rise to a unique domain is known exactly [8]. Using this result, we obtain the allele frequency spectrum for wavelike growth in 1D ($\mu > 2$) as

$$f_{\mathrm{w}}(x) = \left(\frac{L}{\sqrt{2}\chi}\right)^2 p\left(\frac{Lx}{\sqrt{2}\chi}\right), \tag{A5}$$

where $\chi = \sqrt{v/2u}$ is the characteristic length scale for domains growing with front speed v, and

$$p(s) = \frac{\sqrt{\pi}}{4}(1-\mathrm{erf}(s))\left[\sqrt{2\pi}e^{\frac{s^2}{2}}\left(s^2+1\right)\mathrm{erf}\left(\frac{s}{\sqrt{2}}\right)+2s\right], \tag{A6}$$

where erf is the error function. The result is valid as long as the domain sizes are not limited by the range size, i.e. $L \gg \chi$.

The front velocity for arbitrary $\mu > d+1$ is not known analytically, but its limiting value for very large μ in the lattice model is known. In the limit $\mu \gg d+1$, practically all attempted jumps land exactly one lattice site away from the source (this is the lower cutoff for allowed jump distances). Isolated domains grow *via* jumps from the demes situated at the edges, only half of which are successful in advancing the front (the other half land on the occupied side of the front and have no effect). Therefore, the front velocity is $1/2$ a lattice site per generation in the large-μ limit. The frequency spectra for $\mu > d+1$ approach this limit as μ increases, see Fig. 6. We can also extract the μ-dependent front speed by a one-parameter fit of Eq. A5 to the observed frequency spectra, and obtain consistent results when performing fits at different

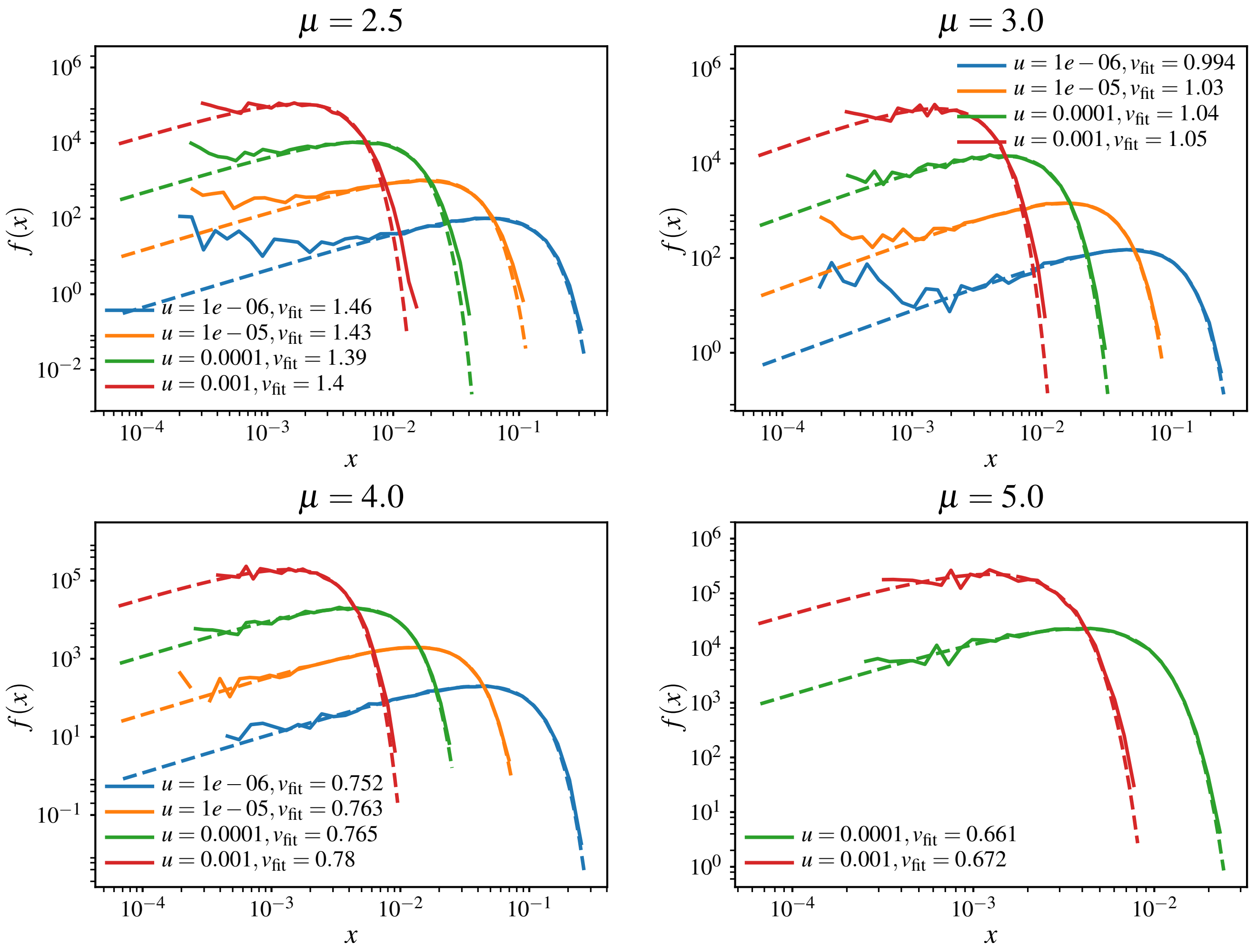

FIG. A3. **Fits to the exact frequency spectrum in the wavelike growth limit.** The measured allele frequency spectra from 1D simulations in the wavelike growth regime ($\mu > d+1$) are shown along with the theoretical form from Eqs. A5–A6. The unknown front speed v is extracted using a one-parameter nonlinear fit, and reported in units of lattice steps per generation. The fit values are consistent with v being determined by μ and independent of $\tilde{u}$. The front speed approaches the limit of 1/2 lattice steps per generation as μ increases.

values of the mutation rate for any given μ, as shown in Fig. A3.

E. DETERMINISTIC APPROXIMATION TO ALLELE FREQUENCY SPECTRA IN 1D

The analysis of the panmictic limit in the main text revealed that the distribution of alleles as $\mu \to 0$ was identical to that of Hoppe's urn process. The continuous-time analogue of Hoppe's urn is the Yule process with immigration, in which new alleles enter the population as a Poisson process with rate θ, and already-present individuals give birth to offspring at rate 1 without death. Yule's process generates the same distribution of allele sizes as Hoppe's urn, but the continuous-time description has the advantage that the dynamics of different alleles are independent: the population of allele i at time t is proportional to e^{t-t_i} where t_i was the time at which it entered the population. Statistical properties of the allele frequencies, such as the frequency spectrum $f_\infty(x)$, can be derived efficiently within this viewpoint.

In our simulations, the growth rate of alleles is *not* constant over time even if we assume panmictic migration; the success of each birth event is proportional to the wild-type fraction w which falls as the simulation progresses. However, as we saw in the main text, the mapping to Hoppe's urn/Yule process remains exact because the rate of generation of new alleles is also proportional to w and the relative rates of birth and migration remain constant throughout the duration of the simulation in the panmictic limit. This is no longer true for $\mu > 0$ when domains grow somewhat contiguously, because the likely targets for migrants become correlated with the occupancy of the lattice and the reduction in growth rate may not simply be given by the fraction w. If we ignore these correlations,

we arrive at the following approximate continuous-time model for the establishment and growth of mutant clones: new alleles enter the population at a constant rate θ, and grow according to the growth rule $\ell(t)$ for the particular dispersal kernel, without interference from other clones.

We can make analytical headway if we further assume that the arrival of new alleles is deterministic rather than Poisson: the kth allele enters the population at time $t_k = k/\theta$, and hence the size of the kth clone is $n_k = \ell(t-k/\theta)$. The total number of alleles, K, is fixed by the range size: $N = \sum_{k=1}^{K} n_k$. In this deterministic model, the strict time ordering of alleles implies that there are k alleles with size greater than or equal to n_k; i.e. if we can invert the n_k relation to get $k(n_k)$, this is the survival function associated with the probability distribution of n_k and hence $x = n_k/N$. The probability distribution of x is precisely the allele frequency spectrum up to a normalization.

Below, we summarize the outcome of computing $f(x)$ according to this deterministic approximation upon using the asymptotic functional forms for $\ell(t)$ in the different regimes in 1D, summarized in Table I.

i. Power-law growth

The deterministic approach can be used to compute an approximate frequency spectrum for the growth form $\ell(t) = At^{1/(\mu-1)}$, which is the asymptotic growth rule for $1 < \mu < 2$. In this case, we have a frequency spectrum that decays as a power law: $f(x) \sim x^{\mu-2}$, up to a hard cutoff at a maximal value determined by the value of K that fills the entire range. Furthermore, the form admits a rescaling that ought to collapse frequency spectra across different system sizes and mutation rates: $f(x) = (L/X_{\text{ave}})^2 F(Lx/X_{\text{ave}})$, where $F(y) = y^{\mu-2}$ up to the cutoff $y_{\max} = \mu/(\mu-1)$, which is the same as Eq. 4 in the main text. Fig. A4 shows that the collapse works very well across different mutation rates and two system sizes. The predicted power law for $f(x)$ is near-quantitative for all μ except $\mu = 1.2$, which is too close to the marginal case $\mu = 1$ for the asymptotic growth rule to be relevant. The predicted cutoff frequency captures the rough location of the dropoff in $f(x)$, but the deterministic approximation fails to capture the "soft shoulder" or the clones at very large frequency, which may have an outsize influence on sampling statistics.

Note that the deterministic approximation predicts a flat frequency spectrum $f(x) = \text{const.}$ for linear growth $\ell(t) = vt$, whereas the exact result for wavelike growth in 1D from the Axe and Yamada results, which we have seen to be quantitatively accurate for $\mu \gg 2$, predict a linear increase in the power spectrum $f(x) \propto x$ for small x. The difference is due to the fact that the deterministic approximation assumes that growth happens symmetrically toward both the left and the right at all times, whereas the wavelike growth limit is characterized by the left and right edges of the domain being interrupted independently as they run into other domains, so that one edge always advances for longer than the other. We can also explicitly include the $\log t$ correction to linear growth exactly at $\mu = 2$, and we find that the low-x behaviour is unaffected (i.e. $f(x) \sim \text{const.}$ as $x \to 0$) but there are contributions at higher x. These arise in the "shoulder" region of the spectrum, which is not captured by the deterministic analysis.

ii. Marginal growth

If we use the growth form for $\mu = 1$ in the deterministic calculation, we no longer get a simple power law for $f(x)$; the functional form is instead $f(x) \sim \exp(\sqrt{a + b\log x}/\sqrt{a + b\log x}/x)$ where a and b depend on the prefactors associated with $\ell(t)$ and on θ and K. This form is not a strict power law in x. However, when the various coefficients are computed using the full expression for $\ell(t)$ measured from the growth of single domains (Appendix A), we find that $f(x)$ behaves similar to a power law over a wide range of n_k, with an effective exponent between -0.65 and -0.85. Using the same rescaling as for the power-law growth for the measured $f(x)$ gives reasonable collapse over a range of values of u and L (Fig. A5) with a power law decay $f(x) \sim x^{-0.72}$. We note that $f(x)$ measured from simulations appears closer to a power-law form for $x \to 0$ than the deterministic approximation.

iii. Stretched exponential growth

In the stretched-exponential growth regime $\mu < d$, the rescaling of the frequency spectra for a specific kernel proposed in Equation 4 is no longer exact. The rescaling assumed that χ set all length scales in the problem; this was true for power-law growth because the halo-dependent scales ψ and ζ were proportional to χ (with proportionality factors that depended only on μ and not on χ). By contrast, for stretched-exponential growth the additional length scales depend on the average clone sizes and hence on $\tilde{u}$. However, Fig. 6 showed that the rescaling captured much of the variation in $f(x)$ across two well-separated mutation rates, down to $\mu = 0.4$.

Although we could compute approximate frequency spectra using the deterministic calculation outlined above, they are less revealing in this regime. Instead, we gauge the inaccuracy of the proposed scaling in the panmictic limit $\mu \to 0$ where we know the exact frequency spectrum f_∞. When $N\tilde{u} = \theta \gg 1$, we have $X_{\text{ave}} \approx -1/(\tilde{u}\log\tilde{u})$ in the panmictic limit. Using this result and the form for f_∞ in Eq. 4, we find that

$$F_\infty(y) = \frac{-1}{y\log\tilde{u}}\left(1 + \frac{y}{\theta\log\tilde{u}}\right)^{\theta-1} \approx \frac{-1}{y\log\tilde{u}}\left(1 + \frac{y}{\log\tilde{u}}\right), \quad \text{(A7)}$$

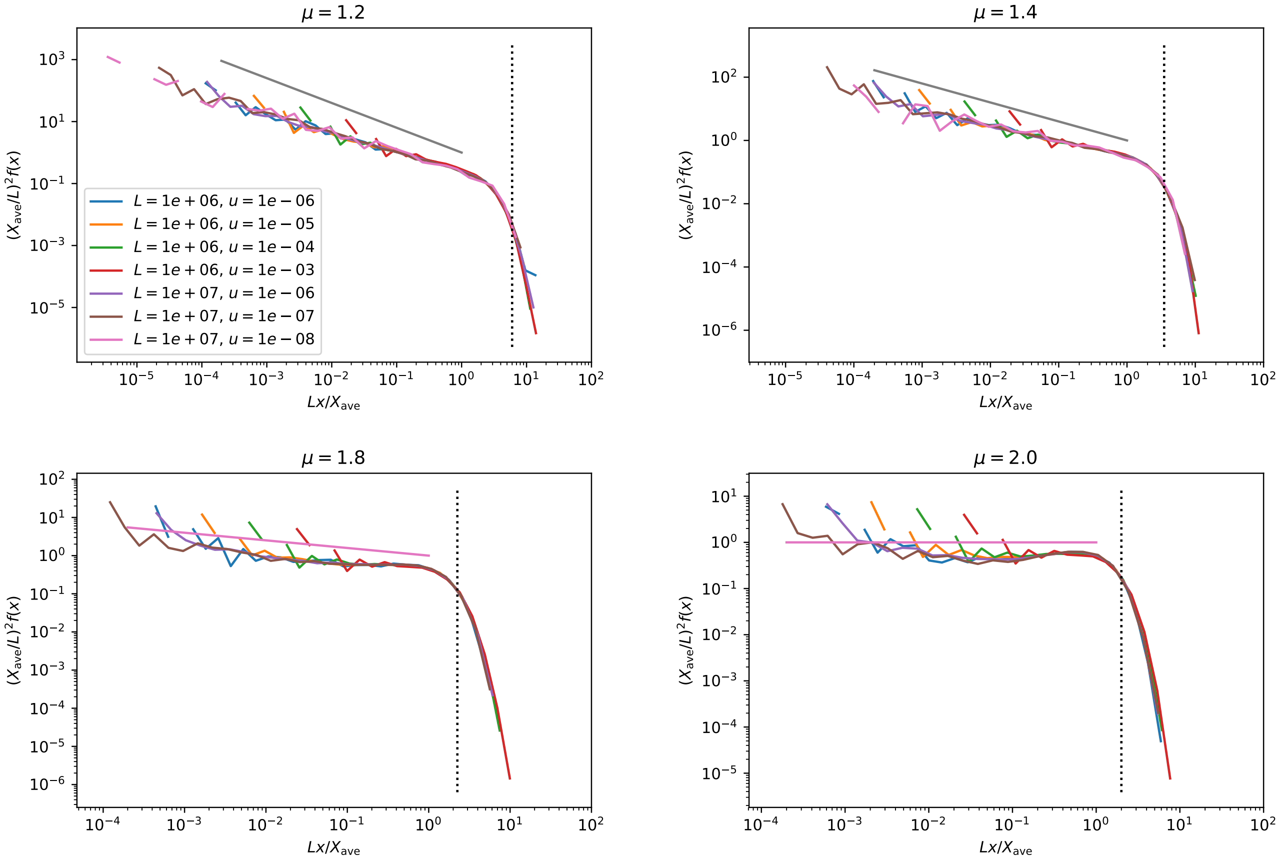

FIG. A4. **Deterministic approximation to allele frequency spectra.** Allele frequency spectra in the power-law growth regime for different mutation rates and system sizes. The rescaling is suggested by the deterministic calculation, it corresponds to a clone size distribution whose only scale is the characteristic length scale χ or equivalently the average clone size $X_{\rm ave}$. The solid line is the prediction $f(y) = y^{\mu-2}$ and the vertical dashed line indicates the maximal rescaled allele frequency $\mu/(\mu-1)$ from the deterministic approximation.

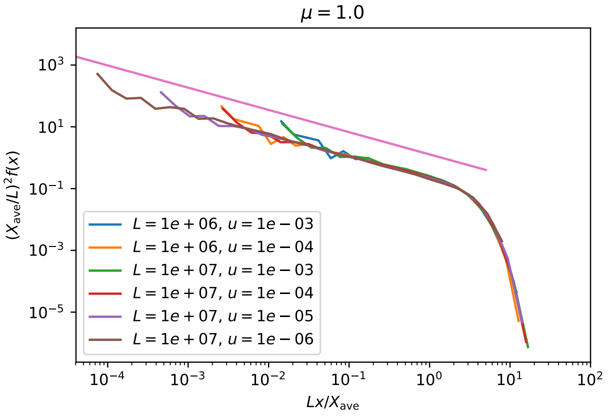

FIG. A5. **Comparison of allele frequency spectra over a range of system sizes for $\mu = 1$.** Allele frequency spectra for $\mu = 1$ over different values of L and u, rescaled according to the assumption that the only length scale for the domains is χ. The low-frequency behaviour is consistent with a power-law decay that goes as $x^{-0.72}$ (straight line).

where $y = Nx/X_{\rm ave}$ and we have used $\theta \gg 1$ in the second step. We find that the function after rescaling has a residual dependence on $\log \tilde{u}$, both in the overall magnitude and in the value $y_{\rm c} \sim \log \tilde{u}$ of the dropoff in f. The gentle logarithmic correction implies that the proposed rescaling still captures much of the variation with mutation rates for a given kernel, even if $\tilde{u}$ is varied by orders of magnitude, thus explaining the decent collapse of curves at different mutation rates in Fig. 6 even for $\mu < d$.

F. ALLELE FREQUENCY SPECTRA WITH A HARD CUTOFF

The measured allele frequency spectra display a power-law behaviour $f(x) \sim x^p$, $p > -1$ for small values of x. For cores growing as contiguous domains, balancing growth and mutation rates gives rise to a characteristic linear domain size χ (and corresponding clone size χ^d) for domain growth before a cone encounters a new mutation. In a finite range of size L^d, such growth would

imply an upper bound on the allowed allele frequency at some value $x_c \sim (\chi/L)^d$. These observations suggest the ansatz for the allele frequency spectra introduced in the main text:

$$f(x) = \begin{cases} \dfrac{p+2}{x_c^{p+2}} x^p, & x < x_c \\ 0, & x > x_c, \end{cases} \tag{A8}$$

where the prefactor is determined by the normalization condition $\int_0^1 x\, f(x) dx = 1$.

This ansatz ignores contributions from higher-frequency clones, which are clearly significant especially for small values of μ. We can evaluate the significance of these contributions by comparing measured quantities to expectations from the hard-cutoff ansatz below.

The average clone size $X_{\text{ave}} \equiv N/n_c = N/\int f(x)dx$ can be evaluated for all $p > -1$ as

$$X_{\text{ave}} = \frac{p+1}{p+2} N x_c. \tag{A9}$$

The sampling probability of observing only one allele in a sample of size j evaluates to

$$P_{\text{hard}} = \int_0^1 x^j f(x) dx = \frac{p+2}{p+j+1} x_c^{j-1} \tag{A10}$$

which deviates weakly from the exponential falloff $P_{\text{hard}} = x^{*j-1}$ expected if all clones are of the same size and hence the same frequency x^*.

G. SAMPLING STATISTICS IN PANMICTIC AND 1D WAVELIKE GROWTH LIMITS

In the panmictic limit, $\mu \to 0$, sampling probabilities are known analytically for all sample sizes [4]. Using $f_\infty(x)$ in Eq. 6 gives $P_{\text{hard}} = \theta(j-1)!\Gamma(\theta)/\Gamma(j+\theta)$ [2, 4] (where Γ denotes the gamma function). The result has two distinct behaviours depending on the value of $\theta = N\tilde{u}$. When $\theta \gg 1$, an exponential falloff $P_{\text{hard}} \sim (1/\theta)^j \theta\Gamma(\theta)$ is recovered for large j, whereas for $\theta \ll 1$, $P_{\text{hard}}(j)$ falls slower than $1 - \theta \log j$.

For 1D wavelike growth with constant front velocity, Ref. [8] provides the exact form for the allele frequency spectrum, Eqs. A5–A6. The probability of observing only one allele in a random sample of size j is then $P_{\text{hard}} = \int_0^1 x^j f(x)\, dx = (\sqrt{2}\chi/L)^{j-1} \int_0^{L/(\sqrt{2}\chi)} s^j p(s)\, ds$. The latter integral cannot be evaluated in a closed form, even when we consider $L/\chi \gg 1$ so that the upper limit can be replaced by $s = \infty$. However, by tracking the position of the maximum value of the integrand which occurs at $s \approx \sqrt{j}$, and using Laplace's method to approximate the integral, we arrive at $\int_0^\infty s^j p(s)\, ds \approx 2j^{j/2} p(\sqrt{j})$, which provides a correction to the leading contribution $(\sqrt{2}\chi/L)^{j-1}$ to P_{hard}. The resulting approximate expression,

$$P_{\text{hard}} \approx 2(\sqrt{2}\chi/L)^{j-1} j^{j/2} p(\sqrt{j}),$$

is used in the dash-dotted line in Fig 7 of the main text. Note that the approximation is only valid when the maximum value of the integrand lies below the upper integration limit; i.e. for $j < L^2/(2\chi^2)$. For larger values of j, P_{hard} is dominated by the upper limit, and scales as $(\sqrt{2}\chi/L)^{j-1} \times (L/(\sqrt{2}\chi))^j p(L/(\sqrt{2}\chi))$ which is independent of j; i.e. the probability of detecting a hard sweep ultimately levels off for sufficiently large j.

[1] O. Hallatschek and D. S. Fisher, Proceedings of the National Academy of Sciences **111**, E4911 (2014), arXiv:arXiv:1403.4639v1.

[2] P. S. Pennings and J. Hermisson, Molecular Biology and Evolution **23**, 1076 (2006).

[3] F. M. Hoppe, Journal of Mathematical Biology **20**, 91 (1984).

[4] W. J. Ewens, Theoretical Population Biology **3**, 87 (1972).

[5] G. Watterson, Theoretical Population Biology **7**, 256 (1975).

[6] P. Ralph and G. Coop, arXiv preprint arXiv:1005.0554v1 (2010).

[7] P. Ralph and G. Coop, Genetics **186**, 647 (2010), arXiv:1005.0554.

[8] J. D. Axe and Y. Yamada, Physical Review B **34**, 1599 (1986).
