# Supplementary figures and images for: Spatial soft sweeps: Patterns of adaptation in populations with long-range dispersal
Source: PLoS Genet. 2019 Feb 11;15(2):e1007936. doi: 10.1371/journal.pgen.1007936 (PMC6386408; doi:10.1371/journal.pgen.1007936)

(a)  $\mu = 0.4$ 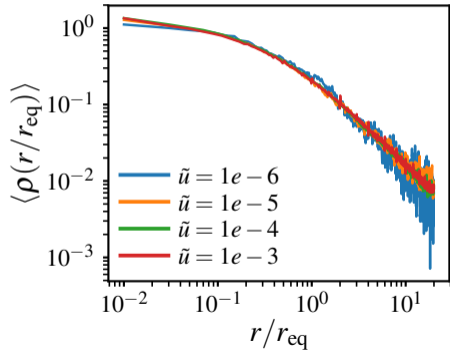(b)  $\mu = 1.0$ 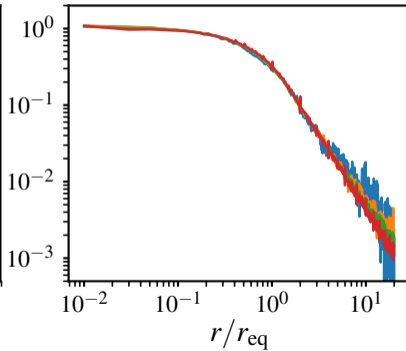(c)  $\mu = 1.6$ 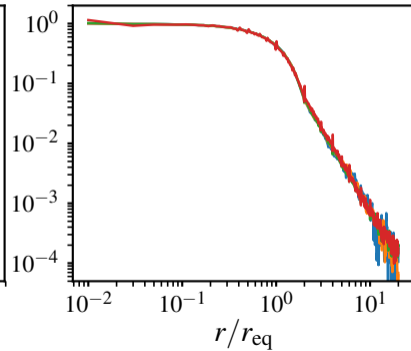

Supplement: S1 Fig — Averaged occupancy profiles 〈ρ〉(r/req) measured from the final states of 1D simulations with L = 106. Panels correspond to different dispersal kernels quantified by μ = 0.4 (a), μ = 1 (b), and μ = 1.6 (c). Colors indicate different rescaled mutation rates. Each curve is itself an average over clones of different sizes, and the average clone sizes vary by orders of magnitude among the different values of u˜. Despite this variation, the profiles for a given dispersal kernel collapse onto a single curve, confirming the validity of the rescaling of the distance variable r with the mass-equivalent clone radius req. The smallest and largest average clone sizes (at u˜=1e-3 and u˜=1e-6 respectively) are (130, 5.8 × 10^4^) for μ = 0.4; (84, 1.6 × 10^4^) for μ = 1.0; and (56, 4100) for μ = 1.6. (PDF) [file pgen.1007936.s004.pdf]
